# Association between trust in the incumbent president and COVID-19 preventive behaviors during the pandemic in four Latin American countries

**DOI:** 10.1590/0102-311XEN023824

**Published:** 2025-02-07

**Authors:** Juliana Mejía-Grueso, Gloria Isabel Niño-Cruz, Javiera Alarcón-Aguilar, Pablo Roa-Urrutia, Sergio Mauricio Moreno-López, Adriano Akira Ferreira Hino, Alexandre Augusto de Paula da Silva, Fernando López, Deborah Salvo, Rodrigo Siqueira Reis, Guillermo Rosas, Andrea Ramírez-Varela

**Affiliations:** 1 Facultad de Medicina, Universidad de los Andes, Bogotá, Colombia.; 2 Escola Superior de Educação Física, Universidade de Pernambuco, Recife, Brasil.; 3 Escola de Medicina e Ciências da Vida, Pontifícia Universidade Católica do Paraná, Curitiba, Brasil.; 4 Independent researcher, Santiago, Chile.; 5 Department of Kinesiology and Health Education, The University of Texas at Austin, Austin, U.S.A.; 6 Prevention Research Center, Brown School at Washington University in St. Louis, St. Louis, U.S.A.; 7 Department of Political Science, Washington University in St. Louis, St. Louis, U.S.A.; 8 School of Public Health, University of Texas Health Science Center at Houston, Houston, U.S.A.; 9 Center for Health Equity, University of Texas Health Science Center at Houston, Houston, U.S.A.; 10 McGovern Medical School, University of Texas Health Science Center at Houston, Houston, U.S.A.

**Keywords:** COVID-19, Pandemics, Behavior, Trust, COVID-19, Pandemias, Conducta, Confianza, COVID-19, Pandemias, Comportamento, Confiança

## Abstract

The COVID-19 pandemic revealed disparities in policy responses in Latin America. We examined the association between trust in the president and COVID-19 preventive behaviors in Brazil, Chile, Colombia, and Mexico. We used data from the *Collaborative COVID-19 Response Survey* by the McDonnell Academy at Washington University in St. Louis (United States), from September 2020 to March 2021. Nonprobabilistic sampling included adult citizens from the four countries. Multivariate negative binomial regression models were applied. The study included 8,125 participants, with Brazil showing the lowest adherence to preventive behaviors (65.5%). Increased adoption of preventive behaviors was linked with ages 18-26 (aIRR = 1.05; 95%CI: 1.01-1.09), 60 or more (aIRR = 1.10; 95%CI: 1.05-1.15), and high socioeconomic status (aIRR = 1.09; 95%CI: 1.05-1.13). Decreased engagement was linked to participants from Brazil (aIRR = 0.74; 95%CI: 0.71-0.78), Mexico (aIRR = 0.95; 95%CI: 0.92-0.99), basic education (aIRR = 0.75; 95%CI: 0.68-0.84), intermediate education (aIRR = 0.88; 95%CI: 0.85-0.91), low socioeconomic status (aIRR = 0.91; 95%CI: 0.87-0.94), lack of concern about contracting COVID-19 (aIRR = 0.93; 95%CI: 0.88-0.98), and poor knowledge about COVID-19 (aIRR = 0.92; 95%CI: 0.88-0.96). No significant association was found between trust in the president and preventive behaviors. Targeted communication, public education, and improved access to reliable information are crucial for fostering preventive behaviors. Public health practitioners should not overly concern themselves with political rhetoric, as our study suggests that trust in political authorities may not systematically affect compliance with directives.

## Resumen

La pandemia del COVID-19 mostró disparidades ante las respuestas de los gobiernos en América Latina. Se evaluó la relación entre la confianza en el presidente y la adopción de conductas preventivas contra el COVID-19 en Brasil, Chile, Colombia y México. Se utilizaron datos de la *Encuesta de Respuesta Colaborativa al COVID-19* de la Academia McDonnell en la Universidad de Washington en St. Louis (Estados Unidos), de septiembre de 2020 a marzo de 2021. El muestreo no probabilístico estuvo conformado por ciudadanos adultos de los cuatro países. Se aplicaron modelos de regresión binomial negativa multivariables. En este estudio participaron 8.125 personas, y Brasil tuvo la adherencia más baja a las conductas preventivas (65,5%). Una mayor adopción de conductas preventivas se asoció con grupos de edad de entre 18 y 26 años (TIa = 1,05; IC95%: 1,01-1,09), de 60 años o más (TIa = 1,10; IC95%: 1,05-1,15) y de nivel socioeconómico más alto (TIa = 1,09; IC95%: 1,05-1,13). Una menor adopción de estos comportamientos se asoció con participantes de Brasil (TIa = 0,74; IC95%: 0,71-0,78), México (TIa = 0,95; IC95%: 0,92-0,99), de educación básica (TIa = 0,75; IC95%: 0,68-0,84) e intermedia (TIa = 0,88; IC95%: 0,85-0,91), nivel socioeconómico más bajo (TIa = 0,91; IC95%: 0,87-0,94), falta de preocupación por contraer COVID-19 (TIa = 0,93; IC95%: 0,88-0,98) y poco conocimiento sobre el COVID-19 (TIa = 0,92; IC95%: 0,88-0,96). La confianza en el presidente y los comportamientos preventivos no mostraron una asociación significativa. La comunicación dirigida, la educación pública y un mejor acceso a la información correcta son cruciales para promover acciones preventivas. Los profesionales de la salud pública no deben preocuparse demasiado por la retórica política, ya que este estudio muestra que la confianza en estas autoridades no afecta sistemáticamente el cumplimiento de las orientaciones de prevención.

## Resumo

A pandemia de COVID-19 revelou disparidades nas respostas políticas na América Latina. Este estudo examinou a relação entre a confiança no presidente e a adoção de comportamentos preventivos em relação à COVID-19 no Brasil, Chile, Colômbia e México. Utilizou-se dados da *Pesquisa de Resposta Colaborativa à COVID-19* da Academia McDonnell na Universidade de Washington em St. Louis (Estados Unidos) de setembro de 2020 a março de 2021. A amostragem não probabilística incluiu cidadãos adultos dos quatro países. Foram aplicados modelos de regressão binomial negativa multivariada. Ao todo, 8.125 indivíduos participaram do estudo, sendo que o Brasil apresentou a menor adesão aos comportamentos preventivos (65,5%). Maior adoção de comportamentos preventivos foi associada às faixas etárias de 18 a 26 anos (RTIa = 1,05; IC95%: 1,01-1,09), 60 anos ou mais (RTIa = 1,10; IC95%: 1,05-1,15) e maior status socioeconômico (RTIa = 1,09; IC95%: 1,05-1,13). A menor adoção desses comportamentos foi associado a participantes do Brasil (RTIa = 0,74; IC95%: 0,71-0,78), México (RTIa = 0,95; IC95%: 0,92-0,99), de educação básica (RTIa = 0,75; IC95%: 0,68-0,84) e intermediária (RTIa = 0,88; IC95%: 0,85-0,91), menor *status* socioeconômico (RTIa = 0,91; IC95%: 0,87-0,94), falta de preocupação com a contaminação pela COVID-19 (RTIa = 0,93; IC95%: 0,88-0,98) e pouco conhecimento sobre a COVID-19 (RTIa = 0,92; IC95%: 0,88-0,96). Confiança no presidente e comportamentos preventivos não demonstraram associação significativa. Mensagens direcionadas, educação pública e acesso aprimorado a informações corretas são cruciais para promover comportamentos preventivos. Os profissionais de saúde pública não devem se preocupar excessivamente com a retórica política, já que nosso estudo sugere que a confiança nessas autoridades não afeta sistematicamente o cumprimento das diretrizes de prevenção.

## Introduction

The rapid spread of COVID-19 worldwide motivated the World Health Organization (WHO) to declare a pandemic on March 11th, 2020 ^1^. Governments and health authorities implemented measures to mitigate the virus spread, namely: restricting social and physical contact (e.g., closing schools and workplaces, suspending public events, reducing or stopping public transportation and travelling, and social distancing measures), information campaigns, and requiring the use of face masks ^2^ ^,^ ^3^. Early detection and rapid response were some of the critical actions implemented across countries ^4^ ^,^ ^5^.

In Latin America, where the COVID-19 pandemic emerged later, such actions were limited by poor health systems capacity, socioeconomic inequalities, and high poverty rates ^6^ ^,^ ^7^. Consequently, the impact of the pandemic on the region was disproportionately greater compared to other locations. Brazil, Colombia, Chile, and Mexico - among the most populous countries in Latin America - experienced some of the highest figures of confirmed cases and deaths from COVID-19 during 2020-2021 ^8^ ^,^ ^9^ ^,^ ^10^ ^,^ ^11^. Therefore, the outlook throughout the pandemic revealed there was no one-size-fits-all approach, as each government decided to implement strategies recommended by global public health agencies ^12^ to a greater or lesser extent. Some of these policies were successful, but they also had negative effects on people’s rights, commerce, and economic output ^2^ ^,^ ^13^ ^,^ ^14^ ^,^ ^15^.

Poor health infrastructure, limited economic support for vulnerable populations, and capacity to deliver social and health services harmed the pandemic response in Latin America ^16^ ^,^ ^17^ ^,^ ^18^. Despite countries’ efforts to improve their communications, there were continuous changes in the information provided to the population and in the methodologies used to estimate infections, deaths rates, and other relevant indicators. These facts affected transparency and communication, diminishing the confidence of citizens experiencing health and financial difficulties, especially during the early stages of the pandemic ^19^. Uncertainty about the evolution of the COVID-19 crisis persisted throughout the region, making the task of public policy preparedness and response even more complex. The way these responses are prioritized is decisive and strategies must be designed to integrate short- and medium-term mitigation goals ^12^ ^,^ ^20^. In line with this public health emergency, governments took some measures to prevent COVID-19 spread ^16^ ^,^ ^21^ ^,^ ^22^ ^,^ ^23^ ^,^ ^24^. Lockdowns were among the most drastic public health measures implemented because many people were forced to drastically change their daily life activities ^24^ ^,^ ^25^ ^,^ ^26^.

The COVID-19 pandemic showed policymaking deficiencies at all levels of government, but especially raised questions about the vulnerability of health systems to politicization and to mis- and disinformation ^25^ ^,^ ^27^ ^,^ ^28^. There is ample evidence that partisanship influenced how receptive individuals were to governmental directives regarding COVID-19, especially in contexts of high political polarization, like Brazil or the United States. In such scenarios, the rhetoric of incumbent politicians was boosted by the use of ideological cues to promote unhealthy attitudes and behaviors among the population ^29^ ^,^ ^30^ ^,^ ^31^.

Beyond extreme partisanship, prevailing levels of trust in authorities at the onset of the COVID-19 emergency constitute a second potential mechanism that could have complicated efforts to contain the pandemic, as trust in authorities is crucial to promote citizen compliance with public health directives ^32^ ^,^ ^33^ ^,^ ^34^. Whereas extreme partisanship characterizes the attitudes of relatively limited segments of a country’s population, widespread mistrust can lead to massive lack of compliance with public health policy directives. Trust is often conceived as a vast reservoir of goodwill, a long-term positive affective predisposition toward political actors that helps citizens support government action even if they are critical of the government’s short-term performance ^35^ ^,^ ^36^ ^,^ ^37^. About 70% of the population of 58 tracked countries believed that the government was not truthful about COVID-19 ^19^. Around the world, opinions about lockdowns and other measures, like social distancing, were diverse and appeared to be linked to trust in the ability of the incumbent governments to manage the COVID-19 pandemic ^38^.

Thus, we asked if trust in the government was a significant individual-level predictor of willingness to embrace with preventive measures once we consider factors such as sociodemographic characteristics and chronic health conditions. To date, evidence in Latin America of the association between trust in the incumbent president and population behavior and adoption of preventive measures remains scant. Accordingly, this study aimed to examine the association between individuals’ trust in the incumbent president and their adoption of COVID-19 preventive behaviors in Brazil, Chile, Colombia, and Mexico.

## Materials and methods

### Study design and setting

This is a cross-sectional and panel study, based on the *Collaborative COVID-19 Response Survey* sponsored by the McDonnell Academy at Washington University in St. Louis (United States). The survey was designed in three different languages: Latin-American Spanish, Brazilian Portuguese, and English. Participants from Brazil, Chile, Colombia, and Mexico received online survey invites from September 2020 to March 2021. Brazil, Colombia, Chile, and Mexico were selected based on their demographic size (they have the first, second, third, and seventh largest populations in Latin America) ^39^, and on variation in the style of their incumbent presidents (Brazil and Mexico were governed by “populist” presidents, whereas presidents in Colombia and Chile belonged to mainstream political parties). The survey was conducted during a phase in which these countries were in total or partial lockdown and pandemic policies of the government were being implemented. The survey was distributed online via direct email contact.

### Sample

Nonprobabilistic sampling with an automated quota was used to collect answers close to Latin American sociodemographic prevalence rates ^40^. The sample consisted of adult citizens (18 years or older) with online access during 2020-2021 that answered the COVID-19 survey. The company Netquest (https://www.netquest.com) relied on a proprietary panel of about 20,000 people in Brazil, Chile, Colombia, and Mexico and distributed the survey invites via email ^41^.

### Survey description

A self-administered survey, 20 to 30 minutes-long, was used for data collection. The survey contained 38 questions addressing various themes, such as political attitudes, economic behavior, knowledge about the spread of COVID-19, medical expenses, personal economic impact of the pandemic, and opinion on several policy items. Additionally, 26 sociodemographic questions and 19 health-related questions were asked.

### Outcome measurement

#### COVID-19 preventive behaviors

The outcome variable was the number of COVID-19 preventive behaviors adopted. It was obtained from the question: “Have you adopted any of the following COVID-19 preventive behaviors over the past week?”, which included activities such as physical distancing (outdoors, indoors, and at the workplace), avoiding indoor or outdoor social gatherings (without physical distancing or facemasks), avoiding crowds/crowded places, handwashing and/or use of hand sanitizers, avoiding touching eyes/nose/mouth, etiquette coughing/sneezing, staying at home (apart from work), working from home, using face masks, and staying up to date with information on COVID-19.

In addition, the broader indicator of COVID-19 preventive behaviors was decomposed into two variables to examine association with trust in the incumbent president when discriminating between community- and individual-level preventive actions ^42^.

#### Community preventive measures

This outcome variable was the number of COVID-19 community preventive behaviors that individuals adopted which included physical distancing in public (outdoors, indoors, and at the workplace), avoiding indoor or outdoor social gatherings, avoiding crowds/crowded places, and working from home.

#### Personal preventive measures

This outcome variable was the number of COVID-19 personal preventive behaviors that individuals adopted, which included handwashing and/or use of hand sanitizers, avoiding touching eyes/nose/mouth, etiquette coughing/sneezing, staying at home (apart from work), using face masks, and staying up to date with information on COVID-19.

### Independent variables

The variable trust in the incumbent president (yes, neutral, no) was included to capture self-reported trust in the president of the country beyond the positions taken regarding the pandemic. Furthermore sociodemographic variables such as country (Brazil, Chile, Colombia, Mexico); sex (female, male); age groups (young adults [18-26 years], adults [27-59 years], and older adults [60 years or older]) ^43^ ^,^ ^44^; ethnicity (white, black, Indigenous, other); educational level (basic, intermediate, advanced); employment seeking employment status (full-time, part-time, unemployed), and socioeconomic status (low, medium, high) were included.

Health and COVID-19 related variables were included: chronic health conditions (yes, no) to capture respondents with at least one chronic condition (e.g., obesity, chronic kidney disease, chronic obstructive pulmonary disease, diabetes type I and II, among others); perceived vulnerability (yes, no) for those who are concerned about the possibility of contracting COVID-19; knowledge about COVID-19, which was obtained from answers to the following questions: “How confident are you that you know how COVID-19 is transmitted?” and “Are you aware of the current recommendations of your country for preventing COVID-19?”. These questions were reported using a 4-point Likert scale (i.e., highly, somewhat, not much, and not confident; highly, somewhat, not much, and not aware) and were recategorized into dichotomous indicators (highly confident, not confident; highly aware, not aware). These were then arranged to classify participants as having good (i.e., highly confident and highly aware), or poor (i.e., highly confident and not aware; not confident and highly aware; or not confident and not aware) knowledge about COVID-19; and perception about the response to COVID-19, which was obtained from the question: “My municipality/city/town’s government has implemented effective strategies to control the COVID-19 pandemic”. This question was reported using a 4-point Likert scale (i.e., strongly agree, agree, disagree, and strongly disagree) and then arranged to classify participants as having favorable or unfavorable perceptions about the local response to COVID-19.

### Data analysis

Variables of interest were analyzed descriptively by country, considering relative, absolute, and proportional frequencies. Analyses of the association between the COVID-19 preventive behaviors outcomes and demographic, health, and contextual factors were performed using negative binomial regression models with robust standard errors ^45^. The outcome variables were tested for dimension reduction (Supplementary Material 1; https://cadernos.ensp.fiocruz.br/static//arquivo/suppl-e00023824_7550.pdf) ^46^ ^,^ ^47^. Furthermore, the associations between subcategories of the COVID-19 preventive behaviors were also assessed at the community and individual levels using negative binomial regression models with robust standard errors. Poisson regression models were disregarded due to overdispersion in the outcome variables and inadequate model fit (Supplementary Material 2; https://cadernos.ensp.fiocruz.br/static//arquivo/suppl-e00023824_7550.pdf) ^48^.

**Figure 1:**
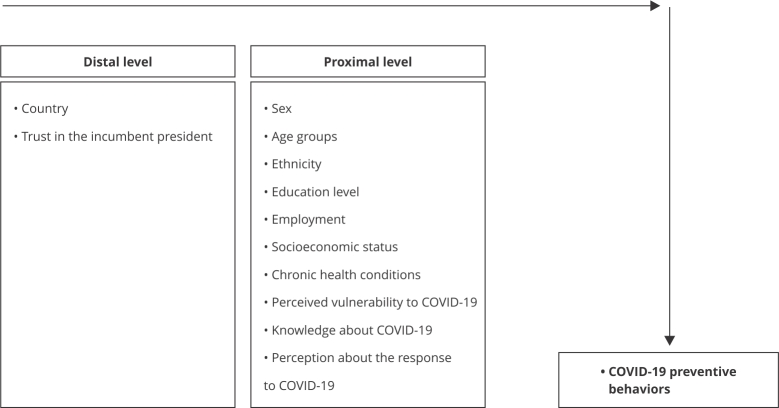
Hierarchical model of COVID-19 preventive behaviors and associated factors.

In the multivariate analyses, the variables from the hierarchical conceptual model (Figure 1) were selected using Kleinbaum et al.’s ^49^ and Greenland’s ^50^ recommendations and those variables with strong associations (p-values < 0.20) remained in the final model. Incidence rate ratios (IRR) and 95% confidence intervals (95%CI) were calculated. Statistical significance was defined as p-values < 0.05. Post-estimation diagnostics were conducted to check for autocorrelation (Durbin-Watson test), multicollinearity (variance inflation factor assessment), and specification assumptions (Supplementary Material 3; https://cadernos.ensp.fiocruz.br/static//arquivo/suppl-e00023824_7550.pdf). All analyses were performed using Stata version 18.0 (https://www.stata.com). 

### Ethics

Ethics approval was obtained from the Institutional Review Boards and Ethics Committees of Washington University in St. Louis (United States, 2020, approval n. 202007185), and Los Andes University (Colombia, 2022, approval n. 202009223).

## Results

### Descriptive analysis by country

The study included 8,125 participants, with a predominant representation of females (51.2%). The distribution across Mexico, Chile, Brazil, and Colombia was well-balanced, with percentages of 52.6%, 52.5%, 50.1%, and 49.5%, respectively (Table 1). Regarding age groups, those aged 27-59 were the majority in all countries (69.3%), and Brazil had the highest proportion (72.7%), followed by Colombia (70.4%), Mexico (67.3%), and Chile (66.8%). Additionally, most participants across all countries identified their ethnicity as “other”.

**Table 1:** Sociodemographic and health characteristics of participants in Brazil, Chile, Colombia, and Mexico (September 2020 - March 2021).

Variables	Total (n = 8,125)	Brazil (n = 1,959)	Chile (n = 2,053)	Colombia (n = 2,064)	Mexico (n = 2,049)
	n (%)	n (%)	n (%)	n (%)	n (%)
Distal level					
Trust in the incumbent president					
Yes	1,986 (24.4)	511 (26.1)	271 (13.2)	478 (23.2)	726 (35.4)
Neutral	971 (12.0)	193 (9.8)	199 (9.7)	284 (13.7)	295 (14.4)
No	5,168 (63.6)	1,255 (64.1)	1,583 (77.1)	1,302 (63.1)	1,028 (50.2)
Proximal level					
Sex					
Female	4,158 (51.2)	982 (50.1)	1,078 (52.5)	1,021 (49.5)	1,077 (52.6)
Male	3,967 (48.8)	977 (49.9)	975 (47.5)	1,043 (50.5)	972 (47.4)
Age group (years)					
18-26	1,499 (18.5)	362 (18.5)	296 (14.4)	405 (19.6)	436 (21.3)
27-59	5,631 (69.3)	1,425 (72.7)	1,372 (66.8)	1,454 (70.4)	1,380 (67.3)
60 or older	995 (12.2)	172 (8.8)	385 (18.8)	205 (10.0)	233 (11.4)
Ethnicity *					
White	2,961 (36.4)	794 (40.5)	951 (46.3)	807 (39.1)	409 (20.0)
Black	296 (3.6)	241 (12.3)	7 (0.3)	40 (1.9)	8 (0.4)
Indigenous	206 (2.5)	18 (0.9)	91 (4.4)	25 (1.2)	72 (3.5)
Other	4,619 (57.0)	899 (45.9)	985 (48.0)	1,184 (57.4)	1,551 (75.7)
Education level **					
Basic	364 (4.5)	232 (11.9)	79 (3.9)	23 (1.1)	30 (1.5)
Intermediate	2,804 (34.5)	937 (47.8)	627 (30.5)	361 (17.5)	879 (42.9)
Advanced	4,870 (59.9)	776 (39.6)	1,298 (63.2)	1,664 (80.6)	1,132 (55.2)
Employment					
Full-time job	3,102 (38.2)	696 (35.5)	742 (36.1)	893 (43.3)	771 (37.6)
Part-time job	1,268 (15.6)	370 (18.9)	268 (13.1)	241 (11.6)	389 (19.0)
Unemployed, seeking employment	3,755 (46.2)	893 (45.6)	1,043 (50.8)	930 (45.1)	889 (43.4)
Socioeconomic status					
Low	2,646 (32.6)	494 (25.2)	988 (48.1)	652 (31.6)	512 (25.0)
Middle	4,022 (49.5)	1,209 (61.7)	798 (38.9)	849 (41.1)	1,166 (56.9)
High	1,457 (17.9)	256 (13.1)	267 (13.0)	563 (27.3)	371 (18.1)
Chronic health conditions ***					
No	3,312 (40.8)	926 (47.3)	668 (32.5)	985 (47.7)	733 (35.8)
Yes	3,555 (43.7)	545 (27.8)	1,135 (55.3)	856 (41.5)	1,019 (49.7)
Perceived vulnerability to COVID-19					
No	783 (9.6)	310 (15.8)	176 (8.6)	186 (9.0)	111 (5.4)
Yes	7,342 (90.4)	1,649 (84.2)	1,877 (91.4)	1,878 (91.0)	1,938 (94.6)
Knowledge about COVID-19					
Poor	1,144 (14.1)	324 (16.5)	330 (16.1)	214 (10.4)	276 (13.5)
Good	6,981 (85.9)	1,635 (83.5)	1,723 (83.9)	1,850 (89.6)	1,773 (86.5)
Perception about the government’s response to COVID-19					
Poor	4,455 (54.8)	920 (47.0)	1,399 (68.1)	1,089 (52.8)	1,047 (51.1)
Good	3,670 (45.2)	1,039 (53.0)	654 (31.9)	975 (47.2)	1,002 (48.9)
Outcome					
COVID-19 preventive behaviors					
Poor	4,289 (52.8)	1,283 (65.5)	1,022 (49.8)	944 (45.7)	1,040 (50.8)
Good	3,836 (47.2)	676 (34.5)	1,031 (50.2)	1,120 (54.3)	1,009 (49.2)
Community preventive measures					
Poor	5,309 (65.3)	1,491 (76.1)	1,324 (64.5)	1,180 (57.2)	1,314 (64.1)
Good	2,816 (34.7)	468 (23.9)	729 (35.5)	884 (42.8)	735 (35.9)
Individual preventive measures					
Poor	4,088 (50.3)	1,174 (59.9)	949 (46.2)	941 (45.6)	1,024 (50.0)
Good	4,037 (49.7)	785 (40.1)	1,104 (53.8)	1,123 (54.4)	1,025 (50.0)

* Missing data = 43 (0.5%);

** Missing data = 87 (1.1%);

*** Missing data = 1,258 (15.5%).

Participants were predominantly of advanced education in most countries (Colombia, 80.6%; Chile, 63.2%; and Mexico, 55.2%), while Brazil had a majority with intermediate education (47.8%). In all countries, the highest proportion of participants were unemployed seeking employment (Chile, 50.8%; Brazil, 45.6%; Colombia, 45.1%; and Mexico, 43.4%). Regarding socioeconomic status, the modal respondent fell into the mid-level category in Brazil (61.7%), Mexico (56.9%), and Colombia (41.1%), whereas a higher proportion in Chile belonged to the low-level category (48.1%). Participants predominantly reported chronic health conditions in Chile (55.3%) and Mexico (49.7%), whereas in Colombia and Brazil, a higher percentage did not report such conditions (47.7% and 47.3%, respectively). Also, in all countries there was a high perceived vulnerability to COVID-19 (Mexico, 94.6%; Chile, 91.4%; Colombia, 91%; and Brazil, 84.2%). Most participants reported a good level of knowledge about COVID-19 (Colombia, 89.6%; Mexico, 86.5%; Chile, 83.9%; and Brazil, 83.5%). Regarding perception of local response to COVID-19, most participants in Chile (68.1%), Colombia (52.8%), and Mexico (51.1%) expressed an unfavorable view, while participants in Brazil (53%) believed their government had effectively adopted measures to contain the pandemic.

Most participants did not trust their country’s president (Chile, 77.1%; Brazil, 64.1%; Colombia, 63.1%; and Mexico, 50.2%). Examining COVID-19 preventive behaviors, a higher portion of participants in Brazil (65.5%) and Mexico (50.8%) demonstrated poor adoption, whereas in Colombia (54.3%) and Chile (50.2%) most showed good adoption to preventive measures. At the community level, participants demonstrated inadequate adoption of these behaviors (Brazil, 76.1%; Chile, 64.5%; Mexico, 64.1%; and Colombia, 57.2%). At the individual-level, most participants in Brazil (59.9%) reported a poor adoption, while in Chile (53.8%) and Colombia (54.4%) the majority described a good adoption of preventive behaviors.

### Multivariate models

#### COVID-19 preventive behaviors

The results of the multivariate analysis indicated that trust in the incumbent president was not significantly associated with COVID-19 preventive behaviors (Table 2). Younger individuals aged 18-26 (adjusted IRR - aIRR: 1.05; 95%CI: 1.01-1.09) and those aged 60 and older (aIRR: 1.10; 95%CI: 1.05-1.15) were more likely to engage in preventive behaviors compared to those aged 27-59. Additionally, individuals from high socioeconomic status (aIRR: 1.09; 95%CI: 1.05-1.13) were more likely to engage in preventive behaviors compared to those from middle socioeconomic status.

**Table 2:** Factors associated with COVID-19 preventive behaviors (n = 8,038).

Variables	Unadjusted IRR	95%CI	p-value	Adjusted IRR	95%CI	p-value
Distal level						
Country						
Brazil	0.69	0.66-0.72	< 0.001	0.74	0.71-0.78	< 0.001
Chile	0.95	0.91-0.98	0.001	0.98	0.96-1.02	0.386
Colombia	1.00			1.00		
Mexico	0.92	0.89-0.95	< 0.001	0.95	0.92-0.99	0.006
Trust in the incumbent president						
Yes	0.95	0.92-0.98	0.058	0.97	0.93-1.01	0.121
Neutral	0.96	0.92-1.00	0.002	0.96	0.91-1.00	0.074
No	1.00			1.00		
Proximal level						
Sex						
Female	0.99	0.96-1.01	0.326	0.99	0.96-1.02	0.461
Male	1.00			1.00		
Age group (years)						
18-26	1.05	1.02-1.09	0.004	1.05	1.01-1.09	0.008
27-59	1.00			1.00		
60 or older	1.08	1.04-1.13	< 0.001	1.10	1.05-1.15	< 0.001
Ethnicity						
White	1.01	0.98-1.04	0.405			
Black	0.81	0.74-0.89	< 0.001			
Indigenous	0.94	0.86-1.03	0.236			
Other	1.00					
Education level						
Basic	0.63	0.57-0.70	< 0.001	0.75	0.68-0.84	< 0.001
Intermediate	0.80	0.77-0.83	< 0.001	0.88	0.85-0.91	< 0.001
Advanced	1.00			1.00		
Employment						
Full-time job	1.00			1.00		
Part-time job	0.97	0.93-1.01	0.180	1.01	0.97-1.06	0.584
Unemployed, seeking employment	0.94	0.92-0.97	< 0.001	0.99	0.96-1.02	0.542
Socioeconomic status						
Low	0.91	0.88-0.94	< 0.001	0.91	0.87-0.94	< 0.001
Middle	1.00			1.00		
High	1.16	1.13-1.20	< 0.001	1.09	1.05-1.13	< 0.001
Chronic health conditions						
No	0.99	0.97-1.01	0.382			
Yes	1.00					
Perceived vulnerability to COVID-19						
No	0.88	0.84-0.93	< 0.001	0.93	0.88-0.98	0.005
Yes	1.00			1.00		
Knowledge about COVID-19						
Poor	0.89	0.85-0.93	< 0.001	0.92	0.88-0.96	< 0.001
Good	1.00			1.00		
Perception about the government’s response to COVID-19						
Poor	1.00			1.00		
Good	0.94	0.91-0.96	< 0.001	0.97	0.94-1.01	0.139

95%CI: 95% confidence interval; IRR: incidence rate ratio.

Note: model adjusted for country, trust in the incumbent president, sex, age groups, education, employment, socioeconomic status, perceived vulnerability to COVID-19, knowledge about COVID-19, and perception about the government’s response to COVID-19.

Note: the preventive behaviors considered are as follows: (1) physical distancing in public (outdoors); (2) physical distancing in public (indoors); (3) physical distancing in public (at the workplace); (4) avoiding indoor or outdoor (without physical distancing or facemasks) social gatherings; (5) avoiding crowds/crowded places; (6) handwashing and/or use of hand sanitizers; (7) avoiding touching eyes/nose/mouth; (8) etiquette coughing/sneezing; (9) staying at home (apart from work); (10) working from home; (11) using face masks; and (12) staying up to date with information on COVID-19.

Several factors were associated with a decrease in the engagement in COVID-19 preventive behaviors. Individuals from Brazil (aIRR: 0.74; 95%CI: 0.71-0.78) and Mexico (aIRR: 0.95; 95%CI: 0.92-0.99) were less likely to engage in preventive behaviors compared to those from Colombia. Those with basic (aIRR: 0.75; 95%CI: 0.68-0.84) and intermediate education (aIRR: 0.88; 95%CI: 0.85-0.91) were less likely to engage in preventive behaviors compared to those with advanced education. Individuals from low socioeconomic status (aIRR: 0.91; 95%CI: 0.87-0.94) were less likely to engage in preventive behaviors compared to those from middle socioeconomic status. Regarding perceived vulnerability, those who were not concerned about COVID-19 infection were less likely to adopt preventive behaviors (aIRR: 0.93; 95%CI: 0.88-0.98) compared to those who were concerned. Lastly, participants with a poor knowledge about COVID-19 (aIRR: 0.92; 95%CI: 0.88-0.96) were less likely to engage in preventive behaviors compared to those with a good level of knowledge.

#### Community preventive measures

There was no significant association between trust in the incumbent president with the adoption of social and physical distancing at the community level (Table 3). Younger individuals aged 18-26 (aIRR: 1.05; 95%CI: 1.02-1.09) and those aged 60 and older (aIRR: 1.10; 95%CI: 1.05-1.15) were more likely to engage in community preventive behaviors compared to those aged 27-59. Also, individuals from high socioeconomic status (aIRR: 1.09; 95%CI: 1.05-1.12) were more likely to engage in community preventive behaviors compared to those from middle socioeconomic status.

**Table 3:** Factors associated with COVID-19 community preventive behaviors (n = 8,038).

Variables	Unadjusted IRR	95%CI	p-value	Adjusted IRR	95%CI	p-value
Distal level						
Country						
Brazil	0.67	0.64-0.71	< 0.001	0.73	0.70-0.77	< 0.001
Chile	0.93	0.91-0.96	< 0.001	0.97	0.94-1.01	0.109
Colombia	1.00			1.00		
Mexico	0.92	0.89-0.95	< 0.001	0.95	0.92-0.99	0.008
Trust in the incumbent president						
Yes	0.94	0.91-0.98	0.001	0.97	0.93-1.01	0.093
Neutral	0.97	0.92-1.01	0.149	0.97	0.92-1.01	0.164
No	1.00			1.00		
Proximal level						
Sex						
Female	0.99	0.97-1.02	0.674	0.99	0.97-1.02	0.720
Male	1.00			1.00		
Age group (years)						
18-26	1.06	1.02-1.09	0.002	1.05	1.02-1.09	0.005
27-59	1.00			1.00		
60 or older	1.08	1.04-1.13	< 0.001	1.10	1.05-1.15	< 0.001
Ethnicity						
White	1.01	0.98-1.04	0.530			
Black	0.79	0.72-0.87	< 0.001			
Indigenous	0.94	0.85-1.04	0.214			
Other	1.00					
Education level						
Basic	0.62	0.56-0.69	< 0.001	0.74	0.67-0.83	< 0.001
Intermediate	0.79	0.76-0.81	< 0.001	0.87	0.84-0.90	< 0.001
Advanced	1.00			1.00		
Employment						
Full-time job	1.00			1.00		
Part-time job	0.98	0.94-1.02	0.394	1.02	0.98-1.07	0.276
Unemployed, seeking employment	0.95	0.92-0.98	0.002	1.00	0.97-1.04	0.872
Socioeconomic status						
Low	0.91	0.87-0.94	< 0.001	0.90	0.86-0.94	< 0.001
Middle	1.00			1.00		
High	1.17	1.13-1.21	< 0.001	1.09	1.05-1.12	< 0.001
Chronic health conditions						
No	0.99	0.96-1.01	0.226			
Yes	1.00					
Perceived vulnerability to COVID-19						
No	0.88	0.83-0.92	< 0.001	0.92	0.87-0.97	0.003
Yes	1.00			1.00		
Knowledge about COVID-19						
Poor	0.89	0.85-0.93	< 0.001	0.92	0.88-0.97	0.001
Good	1.00			1.00		
Perception about the government’s response to COVID-19						
Poor	1.00			1.00		
Good	0.93	0.90-0.96	< 0.001	0.97	0.94-1.00	0.073

95%CI: 95% confidence interval; IRR: incidence rate ratio.

Note: model adjusted for country, trust in the incumbent president, sex, age groups, education, employment, socioeconomic status, perceived vulnerability to COVID-19, knowledge about COVID-19, and perception about the government’s response to COVID-19.

Note: the preventive behaviors considered are as follows: (1) physical distancing in public (outdoors); (2) physical distancing in public (indoors); (3) physical distancing in public (at the workplace); (4) avoiding indoor or outdoor (without physical distancing or facemasks) social gatherings; (5) avoiding crowds/crowded places; (6) staying at home (apart from work); and (7) working from home.

Individuals from Brazil (aIRR: 0.73; 95%CI: 0.70-0.77) and Mexico (aIRR: 0.92; 95%CI: 0.92-0.99) were less likely to engage in community preventive behaviors compared to those from Colombia. Those with basic education (aIRR: 0.74; 95%CI: 0.67-0.83) and intermediate education (aIRR: 0.87; 95%CI: 0.84-0.90) were less likely to engage in community preventive behaviors compared to those with advanced education. Individuals from low socioeconomic status (aIRR: 0.90; 95%CI: 0.86-0.94) were less likely to engage in community preventive behaviors compared to those from middle socioeconomic status. Similarly, those who were not concerned about contracting COVID-19 were less likely to adopt community preventive behaviors (aIRR: 0.92; 95%CI: 0.87-0.97) compared to those who were concerned. Lastly, participants with a poor knowledge about COVID-19 (aIRR: 0.92; 95%CI: 0.88-0.97) were less likely to engage in community preventive behaviors compared to those with a good level of knowledge.

#### Personal preventive measures

For personal preventive measures, individuals with a neutral stance on trust in the incumbent president (aIRR: 0.95; 95% CI: 0.90-1.01) were less likely to adopt these actions (Table 4). Younger individuals aged 18-26 (aIRR: 1.04; 95%CI: 1.00-1.08) and those aged 60 and older (aIRR: 1.09; 95%CI: 1.04-1.14) were more likely to engage in personal preventive behaviors compared to those aged 27-59. Moreover, individuals from high socioeconomic status (aIRR: 1.08; 95%CI: 1.05-1.12) were more likely to adopt personal preventive measures compared to those from middle socioeconomic status.

**Table 4:** Factors associated with COVID-19 personal preventive measures (n = 8,038).

Variables	Unadjusted IRR	95%CI	p-value	Adjusted IRR	95%CI	p-value
Distal level						
Country						
Brazil	0.71	0.68-0.75	< 0.001	0.77	0.73-0.80	< 0.001
Chile	0.96	0.93-1.00	0.027	1.00	0.97-1.04	0.841
Colombia	1.00			1.00		
Mexico	0.92	0.89-0.96	< 0.001	0.95	0.92-0.99	0.006
Trust in the incumbent president						
Yes	0.95	0.92-0.99	0.008	0.97	0.94-1.01	0.184
Neutral	0.94	0.90-0.99	0.015	0.95	0.90-1.01	0.022
No	1.00			1.00		
Proximal level						
Sex						
Female	0.98	0.95-1.00	0.089	0.98	0.95-1.01	0.178
Male	1.00			1.00		
Age group (years)						
18-26	1.04	1.01-1.08	0.018	1.04	1.00-1.08	0.027
27-59	1.00			1.00		
60 or older	1.08	1.03-1.12	0.001	1.09	1.04-1.14	< 0.001
Ethnicity						
White	1.02	0.99-1.05	0.282			
Black	0.84	0.76-0.92	< 0.001			
Indigenous	0.95	0.86-1.04	0.285			
Other	1.00					
Education level						
Basic	0.65	0.58-0.72	< 0.001	0.76	0.69-0.85	< 0.001
Intermediate	0.82	0.79-0.84	< 0.001	0.89	0.86-0.92	< 0.001
Advanced	1.00			1.00		
Employment						
Full-time job	1.00			1.00		
Part-time job	0.96	0.92-1.00	0.053	1.00	0.96-1.04	0.938
Unemployed, seeking employment	0.93	0.91-0.96	< 0.001	0.98	0.94-1.01	0.128
Socioeconomic status						
Low	0.92	0.88-0.95	< 0.001	0.91	0.88-0.94	< 0.001
Middle	1.00			1.00		
High	1.16	1.12-1.19	< 0.001	1.08	1.05-1.12	< 0.001
Chronic health conditions						
No	1.00	0.97-1.02	0.724			
Yes	1.00					
Perceived vulnerability to COVID-19						
No	0.89	0.85-0.94	< 0.001	0.93	0.88-0.97	0.003
Yes	1.00			1.00		
Knowledge about COVID-19						
Poor	0.89	0.85-0.93	< 0.001	0.91	0.87-0.96	< 0.001
Good	1.00			1.00		
Perception about the government’s response to COVID-19						
Poor	1.00			1.00		
Good	0.94	0.92-0.97	< 0.001	0.98	0.95-1.01	0.222

95%CI: 95% confidence interval; IRR: incidence rate ratio.

Note: model adjusted for country, trust in the incumbent president, sex, age groups, education, employment, socioeconomic status, perceived vulnerability to COVID-19, knowledge about COVID-19, and perception about the government’s response to COVID-19.

Note: the preventive behaviors considered are as follows: (1) handwashing and/or hand sanitizers; (2) avoiding touching eyes/nose/mouth; (3) etiquette coughing/sneezing; (4) using face masks; (5) and staying up to date with information on COVID-19.

Meanwhile, Brazil (aIRR: 0.77; 95%CI: 0.73-0.80) and Mexico (aIRR: 0.95; 95%CI: 0.92-0.99) were linked to lower engagement in personal preventive measures compared to Colombia. Individuals with basic education (aIRR: 0.76; 95%CI: 0.69-0.85) and intermediate education (aIRR: 0.89; 95%CI: 0.86-0.92) were less likely to adopt personal preventive measures compared to those with advanced education. Individuals from low socioeconomic status (aIRR: 0.91, 95%CI: 0.88-0.94) were less likely to engage in personal preventive behaviors compared to those from middle socioeconomic status. Additionally, individuals who did not feel at risk of contracting COVID-19 were less likely to adopt personal preventive actions (aIRR: 0.93; 95%CI: 0.88-0.97) compared to those who were concerned. Finally, a poor knowledge about COVID-19 (aIRR: 0.91; 95% CI: 0.87-0.96) was associated with lower engagement compared to good knowledge.

Post-estimation diagnostics for all three models indicated that the models assumptions were satisfactorily met. There was no need of dimension reduction of the outcomes, no significant autocorrelation in the residuals, no multicollinearity issues, and the specification tests indicated that the models provided an adequate fit to the data.

## Discussion

The main findings of the study include: (1) there is no statistically significant association between trust in the incumbent president and the adoption of COVID-19 preventive behaviors; (2) participants in Brazil, Chile, and Mexico did not have high levels of adoption of COVID-19 preventive behaviors, at either the community or individual levels when compared with participants in Colombia; (3) adopting COVID-19 individual and community preventive behaviors was associated with being 18-26 or 60 and older, as well as having high socioeconomic status.

This study findings indicate that there was no clear link between trust in the incumbent president and the practice of COVID-19 preventive behaviors. Prior research in the United States had shown that political factors are intertwined with individuals’ risk perceptions and efforts to reduce those risks ^51^ ^,^ ^52^. This entanglement can create challenges for coordinating public health responses to mitigate a pandemic and population’s adherence to public health interventions ^53^ ^,^ ^54^. A prior study involving 23 countries found that trust in the government was linked to increased handwashing frequency, avoiding crowded spaces, and practicing social isolation or quarantine ^55^. While we did not discover a strong association with preventive measures, our study revealed instead that an individual’s perceived vulnerability to contracting COVID-19 can influence their adoption of effective preventive measures. Consequently, we conclude that public health professionals should not be too concerned about the deleterious effects of “political spin” on their recommendations. Effective messaging requires emphasis on the scientific basis of policy recommendations, with particular emphasis on explaining which conditions put individuals at greater risk of infection.

Participants from Brazil presented the lowest adherence to preventive behaviors for COVID-19. This can be due to various organizational, social, demographic, community, economic, and cultural factors that vary across countries ^18^ ^,^ ^56^. The record indicates that Brazil was the gateway of COVID-19 into Latin America, and despite its late arrival in comparison with other continents, in two months the country quickly reached the highest numbers of cases and deaths from COVID-19 ^57^. In Brazil, each state took charge of organizing its own policies to tackle COVID-19. This scenario led to great differences among states, mainly due to political issues and differential adherence to policy recommendations from the Federal Government. On the other hand, Chile implemented expert-advised measures like border closures, extensive testing, and localized quarantines, avoiding a national lockdown. Colombia declared health emergency early, enforcing strict isolation and banning large gatherings. In addition, Mexico, amid healthcare reform, launched a nationwide campaign emphasizing social distancing and hygiene but faced challenges with limited testing, corruption, and inconsistent government communication ^12^ ^,^ ^16^ ^,^ ^32^ ^,^ ^58^.

The results show association between participants aged 60 years or older and adoption of preventive behaviors. Consistent with other studies ^59^ ^,^ ^60^ ^,^ ^61^, older adults tend to be more likely to take precautionary measures for health and for COVID-19, they are more frail and have a higher level of concern than younger individuals ^62^. In addition, people with high socioeconomic status were more likely to adopt preventive behaviors for COVID-19. These results are consistent with other studies, which highlight that adherence to measures and behaviors related to epidemic prevention in a population can be significantly related to economic factors, such as access to better hygienic conditions, better education, and higher income level ^56^ ^,^ ^63^.

Regarding knowledge about COVID-19, the findings show that people with higher awareness were more likely to adopt preventive behaviors. Existing evidence supports our findings ^59^ ^,^ ^60^ ^,^ ^64^ ^,^ ^65^, since access to education and information about the pandemic encourages people to avoid harmful behaviors and to adopt appropriate actions for better management of the pandemic. Likewise, people who felt more vulnerable were more likely to adopt preventive behaviors. This is consistent with other studies ^64^ ^,^ ^66^ that underscore that the feeling of being more exposed to the virus follows the perception of vulnerability, which drives perceptions about the importance of adopting preventive actions during the COVID-19 pandemic.

We were able to assess the associations between COVID-19 preventive behaviors and related factors and selected predictors that appropriately reflect the multidimensional political and social environments of the studied countries. However, certain limitations must be acknowledged when interpreting the results. The proportion of individuals who consented to participate is limited to those aged 18 and above. Nevertheless, the data revealed a disparity in participation rates between individuals aged 50 and above compared to those aged 23 to 49, likely due to the online nature of the sample collection.

## Conclusion

Our findings shed light on the complex interplay of political dynamics, socioeconomic factors, and individual characteristics in shaping responses to the COVID-19 pandemic in Latin America. Because of the dramatic politicization of responses to COVID-19, particularly in the United States, officials and practitioners may feel pressured to counter ideological narratives concerning public health. Our findings suggest that trust in government is not necessarily a systematic predictor of self-reported behaviors, at least not after controlling for socioeconomic characteristics or chronic health conditions. Admittedly, (lack of) trust in government is only one potential mechanism through which politics might intrude into what should be a science-based disseminating approach. We suspect explicit attempts that surround public health interventions with political counternarratives may be irrelevant or even backfire. Instead, the responses to future pandemics should be country-specific approaches with gender and age considerations to target public health communication to specific demographics, such as younger and older adults, who are more likely to adopt preventive behaviors and help to mitigate risks. Also, governments should invest in public education and information campaigns to increase awareness of the pandemic. Access to accurate information is essential in motivating individuals to adopt preventive actions.
