# Supplementary material for: Association between trust in the incumbent president and COVID-19 preventive behaviors during the pandemic in four Latin American countries
Source: Cad Saude Publica. 2025 Feb 7;41(1):e00023824. doi: 10.1590/0102-311XEN023824 (PMC11805518; doi:10.1590/0102-311XEN023824)
Supplement: Supplementary file 1 [file 1678-4464-csp-41-01-EN023824-s.pdf]

Supplementary Material 1

We performed tests for appropriateness of factor analysis and ran analyses to identify the number of dimensions (or factors) that explain the constructs of 1) COVID-19 preventive behaviors, 2) Community preventive measures, and 3) Personal preventive measures.[1,2]

1) COVID-19 preventive behaviors

The outcome variable was obtained from the following question: "Have you adopted any of the following COVID-19 preventive behaviors over the past week (yes/no)?", which included activities such as physical distancing in public (outdoors, indoors, and at the workplace), avoiding indoor or outdoor (without physical distancing or facemasks) social gatherings, avoiding crowds/crowded places, handwashing and/or hand sanitizers, avoiding touching eyes/nose/mouth, etiquette coughing/sneezing, staying at home (apart from work), working from home, using face masks, and staying up to date with information on COVID-19.

Given that the determinant of the correlation matrix is different from 0 (see Figure 1), the Bartlett's test of sphericity strongly indicates that the variables are intercorrelated, and the variables have a high value of KMO (Kaiser-Meyer-Olkin measure of sampling adequacy), we can do a factor analysis with the variables grouped as COVID-19 preventive behaviors.

```
Determinant of the correlation matrix
Det               =     0.247


Bartlett test of sphericity

Chi-square        =          8016.239
Degrees of freedom =               66
p-value           =             0.000
H0: variables are not intercorrelated


Kaiser-Meyer-Olkin Measure of Sampling Adequacy
KMO              =     0.844
```

Figure 1. Factor test COVID-19 preventive behaviors.

To choose the number of factors to extract, following the Kaiser's rule we extract one factor as in Figure 2 it gives an eigenvalue greater than one in the initial solution.

```
Factor analysis/correlation                    Number of obs    =      8,125
    Method: principal factors                  Retained factors =          1
    Rotation: (unrotated)                      Number of params =         12
```

Factor	Eigenvalue	Difference	Proportion	Cumulative
Factor1	5.29165	4.67848	0.9220	0.9220
Factor2	0.61317	0.24737	0.1068	1.0289
Factor3	0.36579	0.15475	0.0637	1.0926
Factor4	0.21104	0.18408	0.0368	1.1294
Factor5	0.02696	0.05794	0.0047	1.1341
Factor6	-0.03098	0.01010	-0.0054	1.1287
Factor7	-0.04108	0.05826	-0.0072	1.1215
Factor8	-0.09934	0.00835	-0.0173	1.1042
Factor9	-0.10769	0.02358	-0.0188	1.0854
Factor10	-0.13127	0.00053	-0.0229	1.0626
Factor11	-0.13180	0.09548	-0.0230	1.0396
Factor12	-0.22729	.	-0.0396	1.0000

```
LR test: independent vs. saturated:  chi2(66) = 4.6e+04 Prob>chi2 = 0.0000
```

Figure 2. Eigenvalues of factors for COVID-19 preventive behaviors.

Also, the scree plot in Figure 3 with a parallel analysis indicates that we can retain one factor which is above the point of inflexion, and the other factors would explain relatively little additional variation.

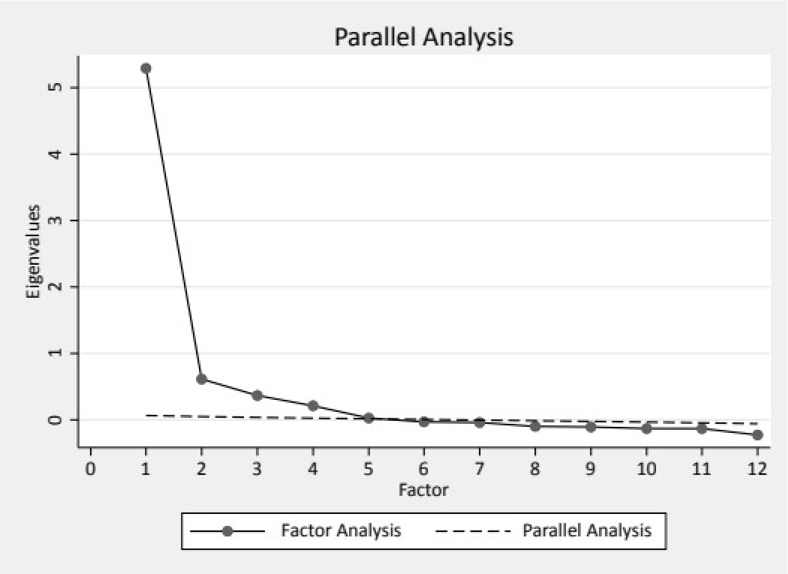

Figure 3. Scree plot with parallel analysis for COVID-19 preventive behaviors.

The factor analysis then suggests retaining only one factor and that the 12 binary variables (preventive behaviors) are all strongly related to a single underlying construct. Then, the variable is the count of COVID-19 preventive behaviors.

2) Community preventive measures

This outcome variable was obtained from the following question: “Have you adopted any of the following COVID-19 preventive behaviors over the past week (yes/no)?” and included the behaviors of physical distancing in public (outdoors, indoors, and at the workplace), avoiding indoor or outdoor social gatherings, avoiding crowds/crowded places, staying at home (apart from work), and working from home.

Given that the determinant of the correlation matrix is different from 0 (see Figure 4), the Bartlett's test of sphericity strongly indicates that the variables are intercorrelated, and the variables have a high value of KMO (Kaiser-Meyer-Olkin measure of sampling adequacy), we can do a factor analysis with the variables grouped as community preventive measures.

```
Determinant of the correlation matrix
Det                =     0.464


Bartlett test of sphericity

Chi-square         =          4438.253
Degrees of freedom =                21
p-value            =             0.000
H0: variables are not intercorrelated


Kaiser-Meyer-Olkin Measure of Sampling Adequacy
KMO               =     0.777
```

Figure 4. Factor test community preventive measures.

To choose the number of factors to extract, following the Kaiser's rule we extract one factor as in Figure 5 it gives an eigenvalue greater than one in the initial solution.

```
Factor analysis/correlation                       Number of obs    =      8,125
    Method: principal factors                     Retained factors =          1
    Rotation: (unrotated)                         Number of params =          7
```

Factor	Eigenvalue	Difference	Proportion	Cumulative
Factor1	3.26883	2.93151	1.0205	1.0205
Factor2	0.33732	0.22118	0.1053	1.1258
Factor3	0.11615	0.17079	0.0363	1.1621
Factor4	-0.05464	0.05885	-0.0171	1.1450
Factor5	-0.11349	0.02852	-0.0354	1.1096
Factor6	-0.14201	0.06705	-0.0443	1.0653
Factor7	-0.20906	.	-0.0653	1.0000

```
LR test: independent vs. saturated:  chi2(21) = 2.4e+04 Prob>chi2 = 0.0000
```

Figure 5. Eigenvalues of factors for community preventive measures.

Also, the scree plot in Figure 6 with a parallel analysis indicates that we can retain one factor which is above the point of inflexion, and the other factors would explain relatively little additional variation.

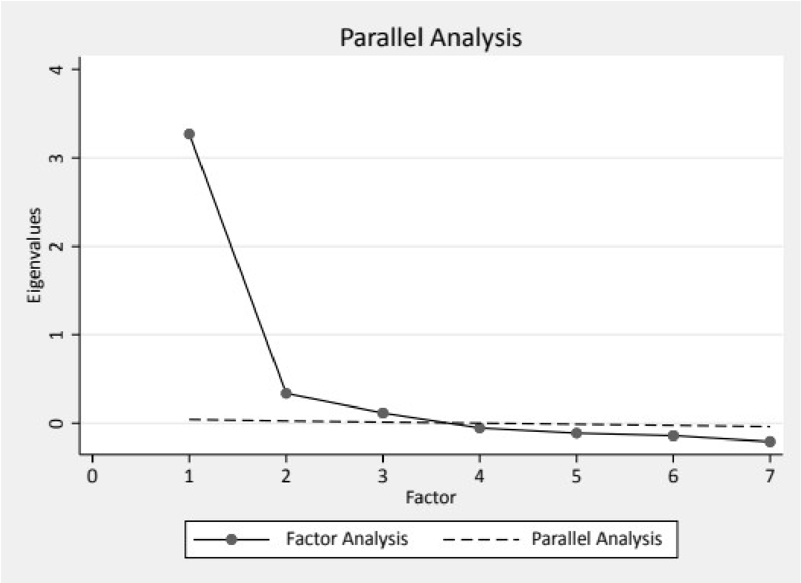

Figure 6. Scree plot with parallel analysis for community preventive measures.

The factor analysis then suggests retaining only one factor and that the 7 binary variables (community preventive behaviors) are all strongly related to a single underlying construct. Then, the variable is the count of community preventive measures.

3) Personal preventive behaviors

The outcome variable was obtained from the following question: “Have you adopted any of the following COVID-19 preventive behaviors over the past week (yes/no)?”, which included behaviors of handwashing and/or use of hand sanitizers, avoiding touching eyes/nose/mouth, etiquette coughing/sneezing, using face masks, and staying up to date with information on COVID-19.
Given that the determinant of the correlation matrix is different from 0 (see Figure 7), the Bartlett’s test of sphericity strongly indicates that the variables are intercorrelated, and the variables have a high value of KMO (Kaiser-Meyer-Olkin measure of sampling adequacy), we can do a factor analysis with the variables grouped as COVID-19 preventive behaviors.

```
Determinant of the correlation matrix
Det                =     0.691


Bartlett test of sphericity

Chi-square         =          2158.831
Degrees of freedom =                10
p-value            =             0.000
H0: variables are not intercorrelated


Kaiser-Meyer-Olkin Measure of Sampling Adequacy
KMO               =     0.703
```

Figure 7. Factor test personal preventive behaviors.

To choose the number of factors to extract, following the Kaiser's rule we extract one factor as in Figure 8 it gives an eigenvalue greater than one in the initial solution.

```
Factor analysis/correlation                     Number of obs    =      8,125
    Method: principal factors                   Retained factors =          1
    Rotation: (unrotated)                       Number of params =          5
```

Factor	Eigenvalue	Difference	Proportion	Cumulative
Factor1	2.50884	2.46371	1.1332	1.1332
Factor2	0.04513	0.10673	0.0204	1.1535
Factor3	-0.06160	0.03835	-0.0278	1.1257
Factor4	-0.09995	0.07842	-0.0451	1.0806
Factor5	-0.17837	.	-0.0806	1.0000

```
LR test: independent vs. saturated:  chi2(10) = 1.6e+04 Prob>chi2 = 0.0000
```

Figure 8. Eigenvalues of factors for personal preventive behaviors.

Also, the scree plot in Figure 9 with a parallel analysis indicates that we can retain one factor which is above the point of inflexion, and the other factors would explain relatively little additional variation.

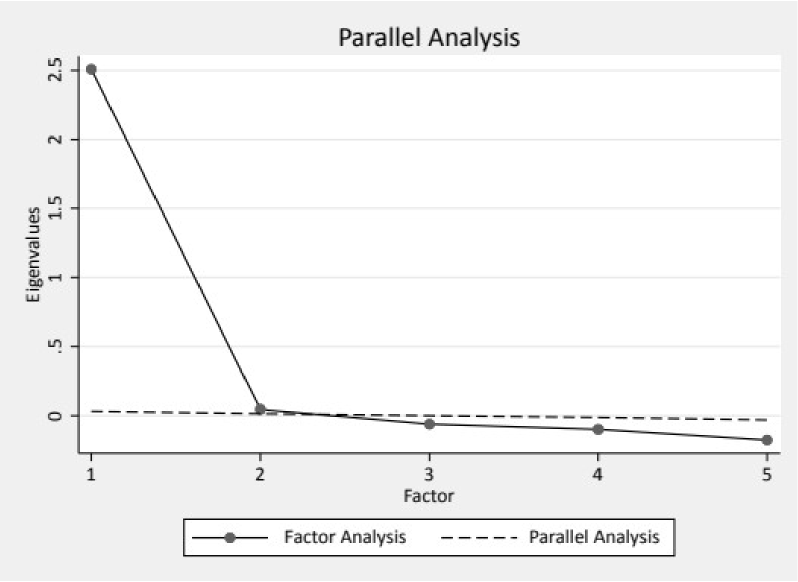

Figure 9. Scree plot with parallel analysis for personal preventive behaviors.

The factor analysis then suggests retaining only one factor and that the 5 binary variables (personal preventive behaviors) are all strongly related to a single underlying construct. Then, the variable is the count of personal preventive behaviors.

Supplementary Material 2

We tested if a Poisson regression was adequate to fit the data for the three models.[3,4]

1) COVID-19 preventive behaviors

```
      Percentiles      Smallest
 1%            0             0
 5%            0             0
10%            0             0       Obs               8,125
25%            0             0       Sum of wgt.       8,125

50%           10                     Mean           7.755323
                        Largest      Std. dev.      4.898874    Deviance goodness-of-fit =  38858.48
75%           12            12                                  Prob > chi2(8027)        =    0.0000
90%           12            12       Variance       23.99896
95%           12            12       Skewness      -.8180224    Pearson goodness-of-fit  =  24670.74
99%           12            12       Kurtosis       1.879008    Prob > chi2(8027)        =    0.0000
```

Figure 10. Summary statistics COVID-19 preventive behaviors and Poisson model goodness-of-fit test.

2) Community preventive measures

```
      Percentiles      Smallest
 1%            0             0
 5%            0             0
10%            0             0       Obs               8,125
25%            0             0       Sum of wgt.       8,125

50%            6                     Mean           4.434462
                        Largest      Std. dev.      2.875908    Deviance goodness-of-fit =  22879.35
75%            7             7                                  Prob > chi2(8027)        =    0.0000
90%            7             7       Variance       8.270846
95%            7             7       Skewness      -.7195253    Pearson goodness-of-fit  =   14822.2
99%            7             7       Kurtosis       1.770864    Prob > chi2(8027)        =    0.0000
```

Figure 11. Summary statistics community preventive measures and Poisson model goodness-of-fit test.

3) Personal preventive measures

```
      Percentiles      Smallest
 1%            0             0
 5%            0             0
10%            0             0       Obs               8,125
25%            0             0       Sum of wgt.       8,125

50%            4                     Mean           3.320862
                        Largest      Std. dev.      2.118083    Deviance goodness-of-fit =  16958.65
75%            5             5                                  Prob > chi2(8027)        =    0.0000
90%            5             5       Variance       4.486277
95%            5             5       Skewness      -.7892042    Pearson goodness-of-fit  =  10831.46
99%            5             5       Kurtosis       1.824069    Prob > chi2(8027)        =    0.0000
```

Figure 12. Summary statistics personal preventive measures and Poisson model goodness-of-fit test.

The distributions of the three outcomes displayed signs of overdispersion—greater variance than might be expected in a Poisson distribution (i.e., mean and variance are the same). In addition, we ran Poisson regression models and the large value for chi-square in the goodness-of-fit and the significant (p-value < 0.05) test statistic indicated that the Poisson models are inappproprite.

Supplementary material 3

We employed a negative binomial regression model to analyze the count data due to observed over-dispersion.[4] In the multivariable analyses, the variables from the hierarchical conceptual model (see Figure 1 manuscript) were selected using Kleinbaum's[5] and Greenland's[6] selection strategy recommendations and those variables with strong associations (p-values < 0.20) remained in the final models.

1) COVID-19 preventive behaviors

Model adjusted for country, trust in the incumbent president, sex, age groups, education, employment, socioeconomic status, perceived vulnerability to COVID-19, knowledge about COVID-19, and perception about the response to COVID-19.

The preventive behaviors considered are as follows: 1) physical distancing in public (outdoors); 2) physical distancing in public (indoors); 3) physical distancing in public (at the workplace); 4) avoiding indoor or outdoor (without physical distancing or facemasks) social gatherings; 5) avoiding crowds/crowded places; 6) handwashing and/or hand sanitizers; 7) avoiding touching eyes/nose/mouth; 8) etiquette coughing/sneezing; 9) staying at home (apart from work); 10) working from home; 11) using face masks; and 12) staying up to date with information on COVID-19.

The dispersion parameter, alpha (α), was estimated to be 0.92, with a robust standard error of 0.33, and a 95% CI [0.86-0.99]. The significant alpha parameter indicates the presence of over-dispersion, justifying the use of the Negative Binomial model over the Poisson model.

Model assumptions: Post-estimation diagnostics indicate that the model assumptions are satisfactorily met. The Durbin-Watson statistic was 1.9, suggesting no significant autocorrelation in the residuals. Variance Inflation Factor (VIF) values for all predictors were below 10, indicating no multicollinearity issues, and the specification test indicates no significant problems (p-value < 0.05) show that the model provides an adequate fit to the data.

2) Community preventive measures

Model adjusted for country, trust in the incumbent president, sex, age groups, education, employment, socioeconomic status, perceived vulnerability to COVID-19, knowledge about COVID-19, and perception about the response to COVID-19.

The preventive behaviors considered are as follows: 1) physical distancing in public outdoors, 2) indoors, and 3) at the workplace); 4) avoiding indoor or outdoor social gatherings; 5) avoiding crowds/crowded places; 6) staying at home (apart from work); and 7) working from home.

The dispersion parameter, alpha (α), was estimated to be 0.50, with a robust standard error of 0.03, and a 95% CI [0.46-0.56]. The significant alpha parameter indicates the presence of over-dispersion, justifying the use of the Negative Binomial model over the Poisson model.

Model assumptions: Post-estimation diagnostics indicate that the model assumptions are satisfactorily met. The Durbin-Watson statistic was 1.9, suggesting no significant autocorrelation in the residuals. Variance Inflation Factor (VIF) values for all predictors were below 10, indicating no multicollinearity issues, and the specification test indicates no significant problems (p-value < 0.05) show that the model provides an adequate fit to the data.

3) Personal preventive measures

Model adjusted for country, trust in the incumbent president, sex, age groups, education, employment, socioeconomic status, perceived vulnerability to COVID-19, knowledge about COVID-19, and perception about the response to COVID-19.

The preventive behaviors considered are as follows: 1) handwashing and/or hand sanitizers; 2) avoiding touching eyes/nose/mouth; 3) etiquette coughing/sneezing; 4) using face masks; 5) and staying up to date with information on COVID-19.

The dispersion parameter, alpha (α), was estimated to be 0.23, with a robust standard error of 0.02, and a 95% CI [0.19-0.27]. The significant alpha parameter indicates the presence of over-dispersion, justifying the use of the Negative Binomial model over the Poisson model.

Model assumptions: Post-estimation diagnostics indicate that the model assumptions are satisfactorily met. The Durbin-Watson statistic was 1.9, suggesting no significant autocorrelation in the residuals. Variance Inflation Factor (VIF) values for all predictors were below 10, indicating no multicollinearity issues, and the specification test indicates no significant problems (p-value < 0.01) show that the model provides an adequate fit to the data.

References

1. Cattell RB. The Scree Test For The Number Of Factors. Multivariate Behavioral Research. 1966 Apr;1(2):245–76.

2. Knekta E, Runyon C, Eddy S. One Size Doesn't Fit All: Using Factor Analysis to Gather Validity Evidence When Using Surveys in Your Research. Brickman P, editor. LSE. 2019 Mar;18(1):rm1.

3. Cameron AC, Trivedi PK. Regression Analysis of Count Data [Internet]. 2nd ed. Cambridge University Press; 2013 [cited 2024 Jul 18]. Available from: https://www.cambridge.org/core/product/identifier/9781139013567/type/book

4. Hardin JW, Hilbe JM. Regression Models for Count Data Based on the Negative Binomial(p) Distribution. The Stata Journal. 2014 Jun;14(2):280–91.

5. Kleinbaum DG, Kupper LL, Nizam A, Muller KE. Applied regression analysis and other multivariable methods. Duxbury Press; 2007.

6. Greenland S. Modeling and variable selection in epidemiologic analysis. American Journal of Public Health. 1989 Mar;79(3):340–9.
